# What the elderly experience and expect from primary care services in KwaZulu-Natal, South Africa

**DOI:** 10.4102/phcfm.v11i1.2100

**Published:** 2019-10-10

**Authors:** Keshena Naidoo, Jacqueline van Wyk

**Affiliations:** 1 Department of Family Medicine, School of Nursing and Public Health, University of KwaZulu-Natal, Durban, South Africa; 2 Discipline of Clinical and Professional Practice, College of Health Sciences, University of KwaZulu-Natal, Durban, South Africa

**Keywords:** geriatric, older adult, primary care, sub-Saharan Africa, KwaZulu-Natal

## Abstract

**Background:**

People aged 60 years and above are predicted to outnumber those aged under 5 years in South Africa for the first time by 2040. This will put increased demands on the health system to address geriatric health needs. However, data on geriatric populations in sub-Saharan Africa are scarce. Health policymakers need to be informed of the expectations of the elderly people regarding health services, especially at primary care level.

**Aim:**

The aim of this study was to explore the experiences and expectations of people aged 60 years and above regarding ageing and health services, and the factors that might improve the quality of primary care services for geriatric patients.

**Setting:**

The study was conducted at three public health primary care facilities in KwaZulu-Natal province, South Africa: one in a rural setting, one in a peri-urban and one in an urban setting.

**Methods:**

This qualitative study involved a purposive sample of 28 participants, aged 60 years and above. Four focus group discussions were conducted in either isiZulu or English, depending on the preference of the participants. Data were analysed thematically using an inductive approach.

**Results:**

Nineteen of the 28 participants were women. Five key findings emerged from the study: (1) long waiting times – participants were distressed by lengthy waiting times, (2) illness-centred care – participants felt that they were seen as diseases to be treated, (3) lack of caring – health providers were perceived to lack compassion, (4) pill burden – participants experienced adverse effects of prescribed medication and (5) need for priority care – participants wanted a separate queue for the elderly.

**Conclusion:**

Health systems and health professions educators should consider the need for patient-centred and integrated care for geriatric populations. Further research is required on the unmet needs of geriatric people in the community.

## Introduction

The geriatric population in sub-Saharan Africa (SSA), which is defined by the World Health Organization (WHO) as people aged 60 years and above, is predicted to increase from 42.6 million in 2010 to 160 million in 2050.^1^ The rapid ageing of populations in this region is attributed to the success of programmes directed at reducing maternal and infant mortality and enhanced access to antiretroviral therapy.^2,3^ In South Africa, the number of people aged 60 years and above is predicted to outnumber those aged under 5 years for the first time by 2040.^4^ Despite the anticipated shift in age-specific health demands, there has been little response from health systems on the continent to address geriatric healthcare. This could be partly because of the lack of data on the health needs of older adults in SSA to inform policymakers.^5^ Most evidence on geriatric health needs is derived from high-income countries (HICs) and thus cannot be generalised to geriatric populations in SSA.

The majority of older adults in SSA utilise primary healthcare facilities for their health needs. Community clinics are the most commonly used providers of health services, while a small number of pensioners also access services from private general practitioners or traditional healers.^6,7^ Specialised geriatric services and specialist geriatricians are rarely available. As reported in 2017, there was only one geriatrician per 275 000 older adults in South Africa.^8^ Despite improved access to primary care services post-democracy, the elderly in South Africa face several challenges in meeting their healthcare needs.^9^ The major barriers that the elderly face in utilising health services include cost, transportation and accessibility. More than 60% of older adults in low- and middle-income countries reportedly did not access healthcare because of the cost of the visit or lack of transportation.^10^ High out-of-pocket expenses are associated with chronic diseases where frequent clinic or hospital visits are required.^9^ Transport is another major barrier to accessing healthcare, especially for the elderly in rural areas and in those with restricted mobility.^11^ Primary care health facilities in KwaZulu-Natal (KZN), South Africa, face resource constraints that present several challenges to geriatric patients. Older adults with physical impairments and urinary incontinence may be discouraged to attend health facilities with long queues and lack of accessible toilets.^9^ Despite policies to improve healthcare access for patients with disabilities, infrastructure at health facilities is still lacking.^12^ Studies in South Africa have reported that elderly patients associated service delivery at the clinics with long waiting times, averaging between 2 and 5 h.^6^ Few facilities prioritise the elderly and physically impaired. Also, existing healthcare services are fragmented with little coordination between care providers.

Currently, the clinical guidelines endorsed for use by primary care providers in South Africa are designed for the management of single diseases, and they inadequately address the problem of multi-morbidity.^13^ Multi-morbidity, defined as the presence of two or more medical conditions, is more prevalent in the elderly people aged 60 and above than in people of other age groups.^14,15^ As a consequence of multi-morbidity and poor coordination of care, polypharmacy in geriatric patients is common. A study conducted by Saka et al. in 2018 described the high prevalence of inappropriate drug prescription for elderly patients in Nigeria and South Africa.^16^ The management of geriatric patients is further compounded by age-related sensory and functional impairments. These impairments could result in poor quality of life and adverse health outcomes in the elderly if not identified and managed appropriately.^6^ Health systems and health professions training assign greater importance to curative medicine than to preventative health and rehabilitation.^17^ Consequently, the complex health needs of geriatric patients are often ignored.

Ageism, defined as the stereotyping or discrimination against people because of their age, is another factor that contributes to the poor quality of healthcare for older adults. A recent study found multiple manifestations of ageism in the healthcare system, ranging from inappropriate treatment to discriminatory communication practices.^18^ A study in Cape Town reported that over 80% of indigent elderly considered staff members at clinics to be unhelpful.^6^ Health professionals with low expectations of health outcomes in the elderly may neglect to address treatable conditions.^19^ Sub-standard clinical care can reinforce negative perceptions of patients towards ageing and the ability to be treated successfully. Evidence indicates that elderly people value a relationship with their healthcare providers based on care for the individual and not focus on disease management.^20^ Training courses for health professionals need to consider a change from traditional disease-focused care to patient-centred care in order to improve the quality of care provided to the elderly.

Little attention has been directed towards health professions training in geriatrics. A survey of medical curricula in SSA revealed little inclusion of geriatrics.^21^ As a result, most medical professionals working in primary care have had little training in geriatric care.^21^ Almost all health professionals in SSA will encounter older adults and will therefore require an understanding of the needs and challenges of geriatric patients. Health professions educators must consider the growing demand of geriatric health services and how to prepare graduates for this. Health policymakers also need to consider how primary care services can be modified to improve the quality of care for the elderly. Given the paucity of literature on the needs of people aged 60 years and above in SSA, this study was conducted to explore the experiences and expectations of older adults regarding ageing and health services, and the factors that might improve the quality of primary care services for geriatric patients.

## Methodology

### Study design

This qualitative exploratory study involved people aged 60 years and above attending primary care facilities in KZN. The interpretative exploratory design was chosen to explore older persons’ perceptions of ageing and age-related health conditions, and their experiences and expectations of health services.

### Population and sampling

A total of 28 participants were purposively selected from four community health centres to reflect the gender and ethnic profile of the population, and to ensure representation from urban, rural and peri-urban communities in KZN. All clinic attendees aged 60 years and above were eligible for the study.

### Demographic data

Of the 28 participants, 19 were women (67.9%). Participants represented different ethnic backgrounds, that is, black, Indian, mixed race and white. The main languages spoken were isiZulu and English.

### Bias

Sampling bias was avoided by carefully selecting participants based on their age, thus representing the group of interest for the research. Interviews were held at a time and place convenient to participants and in a language of their choice. An independent coder was employed to assist in preventing interpretation bias.

### Setting

Data were collected from three health facilities in KZN. These community health centres are managed by the KZN Department of Health. One facility was in a rural location, one in a peri-urban and one in an urban setting. All health facilities from which participants were recruited provide a package of primary care services to the general population and are staffed by medical, nursing and other health professionals.

### Data collection

Four focus group discussions were conducted between October and November 2018. The interviews lasted for about 40–50 min. Two of the four focus group discussions consisted of eight participants each and the other two groups consisted of six participants each. Two of the groups were located in an urban community and one each from a peri-urban and a rural community. The groups were small enough to enable all participants the opportunity to share their insights, but large enough to provide a diversity of understandings. Data were collected on days when the participants were attending the primary care facility for scheduled appointments. Participation in the study did not compromise or facilitate services at the facility. The managers and staff at the facilities assisted the researcher in organising the venue for the interviews and inviting participants to take part. The research assistant explained the purpose of the research and the process of data collection. Interviews were conducted in either isiZulu or English, depending on participants’ preferences. An interview guide was used by a research assistant to collect the data. The discussions were tape-recorded and transcribed verbatim.

### Data analysis

Before data analysis, a research associate fluent and competent in both isiZulu and English translated all isiZulu interview transcripts verbatim into English. The transcripts were imported into Nvivo 12 software. The researcher read through all of the transcripts, together with field notes, and conducted content analysis of each transcript. Similar concepts were clustered together, data were integrated and synthesised into a descriptive structure and codes were created. Themes derived from the codes were categorised into four domains (see Box 1).

BOX 1 Domains of data coding.

**Table:** 

1. Patients’ experiences of ageing and related health condition	2. Patients’ experiences of health services
3. Concerns related to ageing	4. Patients’ expectations of healthcare

### Trustworthiness

The use of an independent coder skilled in the field of research assisted in ensuring dependability. Tape-recorded data and field notes were kept as an audit trail to ensure confirmability.

### Ethical considerations

Participation in the study was voluntary. Written informed consent was obtained from all the participants before the study commenced. All personal data of participants were anonymised. The recordings and transcripts of the focus group discussions were stored on a password-protected laptop accessible only to the researcher and the research assistant. Approval for the study was obtained from the University of KwaZulu-Natal Biomedical Ethics Committee (BE 287/18) and the managers of the health facilities.

## Findings

Five key themes emerged from the data: long waiting times, illness-centred care, lack of caring from professionals, pill burden and the need for priority care. Each theme is discussed below.

### Long waiting times

Participants reported being frustrated by the long waiting times, especially when waiting to see a health professional. It was the most significant concern expressed about visiting health facilities. Multiple visits were sometimes necessary because of shortages of medication:

‘The biggest worry is the queue … sometimes you spend the whole day here.’ (Participant 4, Group 1, 66-year-old female)

‘Sometimes you’re standing in a queue for a long time … over 60 years and you are standing!’ (Participant 4, Group 2, 65-year-old female)

‘All of us have to wait, and they can tell us to come back next day.’ (Participant 1, Group 2, 70-year-old female)

‘We are in our 70s. Imagine … paying all the taxi fare, coming back the next day.’ (Participant 2, Group 1, 72-year-old female)

### Illness-centred care

Health professionals were perceived to view elderly patients as diseases to be treated rather than individuals with health needs. Patients attending the clinic for a chronic disease were not seen as important, and little interest was expressed about patients’ concerns. This reinforced participants’ negative perceptions about chronic illnesses associated with ageing, and significantly altered the identity and self-esteem of participants. Quotes supporting this theme included the following:

‘Every 6 months I must come for my heart, take my medication – I am chronic.’ (Participant 1, Group 1, 66-year-old female)

‘As long as you are chronic, they don’t care …. They just write down your medication and tell you to go. They don’t even look at you. Chronic is just for medication.’ (Participant 3, Group 2, 70-year-old female)

‘If you are a chronic, doctors don’t look at you, just write the prescription.’ (Participant 2, Group 1, 72-year-old female)

‘I try to analyse that word chronic case … it means that you are no more, you will die with it.’ (Participant 5, Group 1, 69-year-old female)

Lack of coordination regarding the various aspects of care needed was also evident. Patients with chronic illnesses received medical services in a designated section of the facility dealing only with patients on chronic medication. If they expressed new symptoms, they would be referred to another department. Participants were confused and frustrated about this fragmentation of services. Participants expressed the following frustrations:

‘The doctor I’m seeing cannot help me, they refer me to the general doctor. What is the purpose of this doctor I’m seeing?’ (Participant 3, Group 3, 68-year-old female)

‘They do not want us to do two things at the same time.’ (Participant 4, Group 3, 66-year-old female)

‘I hear, sometimes not well, but I don’t know where I can get help.’ (Participant 2, Group 4, 74-year-old male)

### Lack of caring from health professionals

Participants perceived health professionals to lack respect or care for older patients. Their lack of interest in patients’ concerns was interpreted as a lack of caring. Patients craved physical contact with the doctor. The need for kindness and empathy was frequently mentioned by most of the participants. This was evident from the following quotes:

‘They do understand but they don’t speak the right way to you, and they wouldn’t do that to their parents.’ (Participant 3, Group 2, 70-year-old female)

‘We worked all our lives … and they feel like they are doing us a favour when you come to a state hospital.’ (Participant 4, Group 2, 65-year-old female)

‘Just be polite, we don’t want to take long.’ (Participant 1, Group 2, 70-year-old female)

‘I think the education has gone so much into them [that] they forgot about love and care.’ (Participant 4, Group 1, 66-year-old female)

‘To be kind, even to ask some questions relating to your life … then you will see he is really taking care of you.’ (Participant 5, Group 1, female)

‘The way he talks can be able to release your pains, your aches.’ (Participant 4, Group 1, 66-year-old female)

### Pill burden

Patients struggled with the multiple medications prescribed or ‘pill burden’. There was poor understanding of the purpose of the medications as health professionals provided little information or education about prescribed treatment. Sometimes, drugs were out of stock and participants were instructed to purchase them at their own cost, or do without. Furthermore, multiple adverse effects were experienced with prescribed medication. Some participants discontinued the medication on their own, while others persisted despite the negative side effects. Participants expressed this in the following ways:

‘Maybe, you have BP and they give you lot of tablets and I do not know what these tablets are for.’ (Participant 3, Group 3, 65-year-old female)

‘I take the tablets but I don’t know where they are helping me.’ (Participant 2, Group 2, 68-year-old female)

‘The doctor gives you tablets without explaining what they are for.’ (Participant 3, Group 2, 70-year-old female)

‘… [*S*]hortage of medicine, now we have to buy medicine. Now we get so less pension money and now you expect us to go buy the tablets.’ (Participant 4, Group 1, 66-year-old female)

‘Another doctor gave me tablets that made my heart beat hard … made me dizzy, I do not drink them and I never went to the doctor. I am right without them.’ (Participant 2, Group 3, 69-year-old female)

### Need for priority care

The participants indicated that they wanted priority care for the elderly, especially for the very old (i.e. over 80 years) and frail patients who attend the facility. Participants in all the groups agreed that there should be a separate queue for the elderly. Quotes supporting this theme included the following:

‘The doctors need to understand we are old, see to us first and not put us with children.’ (Participant 2, Group 1, 66-year-old female)

‘A clinic for old people must be separate.’ (Participant 7, Group 3, 71-year-old male)

‘They should see us first because we are older and do not have the strength.’ (Participant 3, Group 4, 72-year-old female)

## Discussion

This study identified five key findings regarding the perceptions and expectations of people aged 60 years and above about primary healthcare services in KZN province, South Africa. These were (1) long waiting times, (2) illness-centred care, (3) lack of caring from professionals, (4) pill burden and (5) the need for priority care. Participants were dissatisfied with several aspects of primary care services, that is, long waiting times, lack of caring from health professionals, fragmentation of services and multiple medications prescribed.

The main concern expressed was the long waiting times experienced at the clinic. This was exacerbated by return visits to collect medications that were out of stock. Chronic diseases were common among people aged 60 years and above, and necessitated regular clinic visits and multiple medications. The participants found the prescription of multiple medications confusing and sometimes associated with unpleasant side effects. Previous studies have reported that polypharmacy and inappropriate drug prescription are a common occurrence in the elderly. This is associated with an increased risk of adverse drug reactions.^16^ In this study, there was poor communication about the prescribed drugs. Little information was provided in this regard, and there was a lack of knowledge among participants about their medications. As a result, the participants often self-managed the adverse effects experienced with medication without discussing with their healthcare providers. Greater vigilance is required by prescribers when treating the elderly patients.

Clinic services were disease-centred, resulting in fragmentation of the care provided to elderly patients. Participants were required to see different health providers at the same clinic to attend to different health concerns. Participants perceived healthcare professionals to lack respect or care for them, as they showed little interest about patients’ concerns. It was important for the participants that health professionals demonstrate kindness and compassion towards them.

Recommendations emerging from this study to make primary care services more age-friendly included the following: medical staff members need to be more empathetic, clinics should have an integrated service for all health concerns and there should be a priority queue for the elderly and very ill patients. Patient-centred and integrated care for older adults are well-documented principles considered essential for age-friendly services.^22^ The participants were in agreement with these principles. However, despite the economic and health benefits of organising integrated healthcare services for the elderly, there has been little success globally in achieving this objective.^23^ A survey of people over 60 years in 11 HICs reported a lack of coordination of care in 41% of cases.^24^ Countries in SSA need to look at innovative and resource-efficient models of providing quality care to geriatric patients in our context. Collaboration between health professionals is essential to provide comprehensive care to geriatric patients and reduce polypharmacy and inappropriate drug prescription. Integral to this is the inclusion of geriatric care training in health professions education that emphasises patient-centredness and integrated care.

## Limitations

This study was a community clinic-based study and was conducted over a short period of time. It may not represent all people aged 60 years and above in KZN, especially those who do not regularly use health services. Further research is required on the elderly population in the community.

## Conclusion

This study provides data on the quality of primary healthcare services provided to the elderly people in KZN from the perspective of the recipients of care. The challenges experienced by people aged 60 years and above who attend community health facilities included long waiting times, lack of caring from health professionals, high pill burden, illness-centred care and low priority care to the elderly. There was strong support for facilities to have a priority queue to attend to the very old (i.e. above 80 years) and frail patients. The fragmented services reported by the study participants highlighted the need for integrated healthcare services at primary care level. As geriatric patients are prone to multiple health problems, they would benefit from integration of various services required, and there should be an emphasis on function rather than on single-disease treatment.^25,26^ This would potentially reduce waiting times and improve the overall quality of geriatric healthcare. Training of health professionals should consider the need for a compassionate and comprehensive approach when seeing geriatric patients. Greater exposure to geriatric medicine and awareness regarding the unique needs of older adults are critical to health professions training in KZN, South Africa.

The key recommendation for health policymakers and educators is to align geriatric health services with a patient-centred and integrated approach. This study was limited to facility-based care of the elderly in KZN, South Africa. Further research is required regarding the unmet health needs of the elderly in the community. There is also a need for research on health professions education regarding geriatric healthcare, particularly in SSA.
